# The link between neurosteroids and syndromic/syndromal components of the mood spectrum disorders in women during the premenstrual phase

**DOI:** 10.1186/1745-0179-4-3

**Published:** 2008-02-26

**Authors:** Maria Carolina Hardoy, Claudia Sardu, Liliana Dell'Osso, Mauro Giovanni Carta

**Affiliations:** 1 Department of Public Health, Centre for Research and Clinical Practice in Mental Health, Iglesisas, University of Cagliari, Italy; 2 Department of Psychiatry, Neurobiology, Pharmacology and Biotechnology, University of Pisa, Italy

## Abstract

**Objectives:**

Females with a lifetime diagnosis of major mood disorder (Bipolar Disorder BD, Major Depressive Disorder MMD) investigated during the luteal phase of their menstrual cycle and in a condition of clinical well-being showed higher blood serum concentrations of progesterone and allopregnanolone compared to healthy controls. Women with BD presented even higher levels than those affected by MDD. This study attempted to verify, in line with a dimensional approach, if the possible differences in neurohormonal levels may be directly linked to some syndromal clusters (dimensions) of the mood spectrum disorders indipendently of diagnosis.

**Methods:**

Premenstrual concentrations of allopregnanolone, THDOC, progesterone, and cortisol were measured in 3 groups of women: 17 BD and 14 MDD outpatients, and 16 control subjects. Psychiatric evaluation was performed with the SCID-I interview and the SCI-MOODS-SR questionnaire. The correlation between steroid levels and mood disorder syndromal cluster (SCI-MOODS-SR domains and sub-domains) was evaluated by means of analysis of main components with Varimax rotation and Kaiser's normalization (which provided for inclusion of all components with an Eigen value >1).

**Results:**

Analysis of the main components evidenced the presence of 3 components: 1) mania, 2) depression both with mixed component 3) steroid + manic cognitivity and suicidal ideas.

**Conclusion:**

Levels of allopregnanolone and progesterone do not correlate with the association of the depressive and manic syndromes, but rather with mixed symptomatological aspects, and in particular with cognitive manic and depressive (with suicidal thoughts) dimensions. Further studies should be carried out to confirm these findings.

## Introduction

Clinical and preclinical studies have suggested that fluctuations in the peripheral and brain concentrations of progesterone and its metabolites 3α-hydroxy-5α-pregnan-20-one (allopregnanolone) and 3α,21-dihydroxy-5α-pregnan-20-one (THDOC) might play an important role in certain pathological conditions characterized by emotional or affective disturbances, including major depression, anxiety disorders, and schizophrenia [1,2]. Moreover, it has been shown that administration of drugs having clinical relevance in the treatment of these pathologies influence the secretion of these hormones [1,2].

In a recent study, in a sample of female patients with a lifetime diagnosis of major mood disorder (Bipolar Disorder -BD, Major Depressive Disorder -MDD) investigated during the luteal phase of their menstrual cycle and in a condition of clinical well-being, we found higher blood serum concentrations of progesterone and allopregnanolone compared to healthy controls. Moreover, women with BD presented even higher levels than those affected by MDD [3].

Several studies reported a decrease of allopregnanolone in the blood [4], or brain liquor [5] of patients with Major Depression. These studies were all performed on patients during depressive episodes, whilst on the contrary, our patients were in a state of well-being. Moreover, the study carried out by Uzunova [4] reported an increase of plasmatic allopregnanolone levels after treatment with fluoxetine or fluvoxamine, which is paralled to the increase in score at the Hamilton Rating Scale for Depression. In our study, both subjects treated pharmacologically and drug-free cases presented differences in blood serum concentrations of steroids versus control subjects.

In the study of Uzunova [4] no tests were performed to verify whether the increase in allopregnanolone concentrations observed was related to a specific symptomatological component.

The aim of our study was to verify whether neuroactive steroid levels correlate with specific syndromal clusters present throughout the entire spectrum of mood disorders.

Indeed, a continuum between unipolar and bipolar disorders has been hypothesized [6-10], in order to provide a "dialectic" point of view between the traditional "categorial" diagnostic approach and a more innovative "dimensional" approach, syndromic clusters throughout the entire bipolar disorder spectrum, independent of diagnostic subdivision, should be verified to ascentain which are affected by blood serum levels of neuroactive steroids.

In line with a categorial approach, the possible differences in neurohormonal levels may be directly linked to the type of diagnosis (eg. Major Depression, Panic Disorder, Bipolar Disorder), whilst a dimensional approach may vary on the basis of syndromal components present in the "spectrum of disorders" examined.

## Materials and Methods

### Study design and subjects

The sample of the present article is the same of a previous paper above cited [3]. Of course, instruments used and analysis performed for this paper are different. A case-controlled study was performed to compare the plasma concentrations of allopregnanolone, THDOC, progesterone, and cortisol in the premenstrual phase of the menstrual cycle among three groups of women: outpatients with BD, outpatients with MDD, and control subjects. The study was approved by the institutional review board of the "Università Europea del Mediterraneo" and written informed consent was obtained from all subjects.

In an attempt to verify which of the various syndromic mood components showed a tendency to correlate with blood serum neurosteroid levels, both main components and blood serum steroid levels were analysed on the basis of results reported throughout the entire sample at single domains of the SCI-MOODS-SR rating scale.

The study subjects comprised women of reproductive age divided into three groups: 17 women with a lifetime diagnosis of BD I (n = 4, 23.5%) or II (n = 13, 76.5%) according to DSM-IV-TR [11] and in a condition of well-being without relapse or recurrence during the 3 months prior to study enrollment; 14 women with a diagnosis of MDD according to DSM-IV-TR [11] and in a condition of well-being without relapse or recurrence during the 3 months prior to study enrollment; and 16 women with no diagnosis of lifetime psychiatric disorders and with no family history of mood disorders or anxiety. Subjects were enrolled on a consecutive basis on referral to a private psychiatric practice. Personal and anamnestic characteristics of the study subjects are shown in Table 1. All participants were of a similar age. A greater proportion of subjects with a diagnosis of BD took antipsychotic drugs (70.6%) or mood stabilizers (52.9%) compared with those affected by MDD (0 and 0%, respectively), but no signifcant differences either in the use of selective serotonin reuptake inhibitor (SSRI) or tricyclic antidepressants or in the proportion of drug-free subjects were apparent between the two patient groups. Subjects with BD were characterized by a longer course of illness and had been in psychiatric care for a longer period of time compared with those with MDD.

**Table 1:** Personal and anamnestic characteristics of samples.

Samples	Mean Age ± SD	Familiarity Mood disorders	Antipsychotics	SSRIs	Stabilisers	Tricyclic drugs	Drug-free	Years of Illness (mean ± SD)	Years of care (mean ± SD)	Comorbidity Panic Disorder	Comorbidity Obsessive-Compulsive Disorder	Comorbidity Eating Disorder
1. BP (N = 17)	37.2 ± 8.0	11	13	10	9	2	2	14.8 ± 5.7	5.9 ± 2.9	9	6	3
2. MDD (N = 14)	34.4 ± 8.5	6	0	7	0	1	6	8.9 ± 4.5	2.9 ± 2.5	8	4	4
3. Controls (N = 16)	37.1 ± 8.2											
	F = 0.55, (2,44,46 DF)	X2 = 2.3	X2 = 17.9	X2 = 0.7	X2 = 8.0	X2 = 0.1	X2 = 2.4	F = 9.9	F = 9.3	X2 = 0.1	X2 = 0.1	X2 = 0.1
	P = 0.58	NS	P < 0.0001	NS	P < 0.01	NS	NS	P < 0.01 (1,29,30 DF)	P < 0.01 (1,29,30 DF)	NS	NS	NS

Exclusion criteria included comorbidity with invalidating physical illnesses or lesser physical disorders (such as ovarian cysts or immune diseases) that might have affected steroid hormone concentrations, as well as the use of drugs (with the exception of psychotropic agents) that might affect steroid hormone concentrations (such as contraceptive pills and steroids).

Inclusion criteria included regular menstrual cycles and the absence of stressful life events during the previous 3 months.

### Blood sampling and measurement of steroid concentrations

Blood samples were obtained between 0900 and 0930 hours from all study subjects 7 to 10 days before menstruation. All subjects had fasted overnight and had taken absolutely no drugs during the previous 24 h before blood collection. Plasma was isolated from the blood specimens, and 1-mL portions were diluted with 2 mL of water and then subjected to extraction three times with 3 mL of ethyl acetate. The combined organic phases were dried under vacuum. Recovery of steroids during extraction was monitored by the addition of trace amounts of [3H]progesterone (6000 to 8000 cpm, 52 Ci/mmol) and was found to be ~90%. Quantitative analysis of steroids was performed by radioimmunoassay with specific antibodies and respective tritiated standards as described previously [12,13]. Antibodies to allopregnanolone and to THDOC were characterized previously [12] and those to progesterone and to cortisol were from ICN (Costa Mesa, CA).

### Psychiatric assessment

Psychiatric assessment of all subjects was performed by a psychiatrist trained in the use of the Structured Clinical Interview for DSM-IV Axis I Disorders (SCID-I) [14]. Clinical interviews took place at a private medical practice. All participants were subjected to a questionnaire concerning personal and anamnestic details, a self-administered questionnaire on symptoms of Premenstrual Dysphoric Disorder (PMDD) according to DSM-IV [15], a structured clinical interview (SCID-I), and the Structured Clinical Interview for the Spectrum of Mood Disorders, Self Report (SCI-MOODS-SR, self-rating questionnaire, lifetime version) [16-20]. The SCI-MOODS-SR rating scale comprises 161 items and 4 domains (mood, energy, cognitivity and rhythmicity), the first 3 of which each have 2 sub-domains (depressive and manic/hypomanic) [8].

### Statistical analysis

Data were analyzed with the SPSS-SP 12.0 software package (SPSS inc., Chicago Illinois). The correlation between steroid levels, mood disorders (SCI-MOODS-SR domains and sub-domains) and premenstrual symptoms was evaluated by means of analysis of main components with Varimax rotation and Kaiser's normalization (which provided for inclusion of all components with an Eigen value >1).

## Results

The plasma concentrations of cortisol, progesterone, 3α,5α-THPROG, and 3α,5α-THDOC in patients with BD, patients with MDD, and control subjects, as presented in the previous paper [3], are shown in Table 2. The concentrations of progesterone and 3α,5α-THPROG differed significantly among the three groups, whereas that of 3α,5α-THDOC showed a tendency to differ. No significant difference was apparent in the plasma levels of cortisol among the three groups.

**Table 2:** Plasma concentrations of steroids (ng/mL) among patients with bipolar disorder (BP), patients with major depressive disorder (MDD), and control subjects.

Steroid	BD	MDD	Controls	*F *(2, 44, 46)	*P*	*Bonferron BD vs Cs t Sstudent 44DF*	*Bonferroni BD vs MDD t Sstudent 44DF*	*Bonferroni MDD vs Cs t Sstudent 44DF*
Cortisol	127.7 ± 40.4	116.8 ± 54.2	108.4 ± 37.2	0.80	0.456	1.2.	0.5	0.1
Progesterone	11.0 ± 2.6	4.0 ± 3.6	1.0 ± 1.1	64.9	<0.0001	11.0, *P *< 0.0001	7.0, *P *< 0.001	3.2, *P *< 0.0001
3α,5^α ^THPROG	9.6 ± 2.3	5.1 ± 1.2	2.1 ± 0.8	91.7	<0.0001	13.4, *P *< 0.001	7.7, *P *< 0.0001	3.2, *P *< 0.001
3α,5α-THDOC	2.1 ± 3.0	1.1 ± 0.4	0.6 ± 0.4	2.8	0.073	2.1	0.4	0.1

The analysis of main components performed taking as items frequency of response to SCI-MOODS-SR domains and cortisol concentrations, allows 3 components accounting for a total of 70.3% of variance to be identified. Table 3 illustrates the rotation matrix of components and reveals how component 1 (33.7% of total variance, Eigen value 5.9) aggregates SCI-MOODS-SR items:

**Table 3:** Rotation matrix of SCI-MOODS-SR components/cortisol (analysis of main components with Varimax rotation and Kaiser's normalization)

SCI-MOODS-SR DOMAIN	Item	Component 1	Component 2	Component 3
Depressed Mood	D1-28	0.92	0.16	0.08
Manic Mood	D29-57	0.39	0.70	0.13
Depressive Energy	D58-67	0.86	0.13	0.16
Manic Energy	D68-80	0.44	0.74	0.03
Depressive Cognitivity	D81-98	0.75	0.45	0.10
Depressive Cognitivity	D99-108	0.67	0.10	0.43
Manic Cognitivity	D109-124	-0.02	0.79	0.27
Manic Cognitivity	D125-131	0.16	0.69	0.00
Rhythmicity	D132-137	0.32	0.73	-0.13
Rhythmicity	D138-149	0.67	0.36	-0.03
Rhythmicity	D150-161	0.72	0.37	0.13
Cortisol		0.11	0.09	0.91

D1-28, Depressed Mood, (0.92); D58-67, Depressive Energy, (0.86); D81-98, Depressive Cognitivity, (0.75); D150-161, Rhythmicity, (0.72); D99-108, Depressive Cognitivity, (0.67); D138-149, Rhythmicity, (0.67); D68-80, Manic Energy, (0.44); D29-57 (0.39); component 2 (26.7% of variance, Eigen value 3.2) aggregates SCI-MOODS-SR items: D109-124, Manic Cognitivity, (0.79); D68-80, Manic Energy, (0.74); D132-137, Rhythmicity, (0.73); D29-57, Manic Mood (0.70); D125-131, Manic Cognitivity, (0.69); D81-98, Depressive Cognitivity, (0.45); D150-161, Rhythmicity, (0.37); D138-149, Rhythmicity, (0.36); and component 3 (9.8% of total variance, Eigen value1.2) aggregates the level of cortisol (0.91) with SCI- MOODS-SR item: D99-108, Depressive Cognitivity, (0.43).

The analysis of main components performed taking as items frequency of response to SCI-MOODS-SR domains and progesterone concentrations, allows 3 components accounting for a total of 71.2% of variance to be identified. Table 4 illustrates the rotation matrix of components and reveals how component 1 (49.7% of total variance, Eigen value 3.9) aggregates SCI-MOODS-SR items: D1-28, Depressed Mood, (0.91); D58-67, Depressive Energy, (0.86); D81-98, Depressive Cognitivity, (0.73); D99-108, Depressive Cognitivity; (0.71); D150-161, Rhythmicity, (0.67); D138-149, Rhythmicity, (0.65); D29-57, Manic Mood, (0.37); component 2 (27.6% of variance, Eigen value 3.3) aggregates SCI- MOODS-SR items: D132-137, Rhythmicity, (0.75), D125-131, Manic Cognitivity, (0.73); Manic Energy, D68-80 (0.73); D29-57, Man ic Mood (0.72); D109-124, Manic Cognitivity, (0.70); D81-98, Depressive Cognitivity (0.49); D138-149, Rhythmicity, (0.46); D150-161, Rhythmicity, (0.40); and component 3 (9.2% of total variance, Eigen value 1.1) aggregates the level of progestrerone (0.87) with SCI- MOODS-SR items: D109-124, Manic Cognitivity, (0.52); D99-108 Depressive Cognitivity, (0.31).

**Table 4:** Rotation matrix of SCI-MOODS-SR components/progesterone (analysis of main components with Varimax rotation and Kaiser's normalization)

SCI-MOODS-SR DOMAIN	Item	Component 1	Component 2	Component 3
Depressed Mood	D1-28	0.91	0.21	0.04
Manic Mood	D29-57	0.37	0.72	0.10
Depressive Energy	D58-67	0.86	0.13	0.22
Manic Energy	D68-80	0.39	0.73	0.24
Depressive Cognitivity	D81-98	0.73	0.49	0.06
Depressive Cognitivity	D99-108	0.71	0.08	0.31
Manic Cognitivity	D109-124	-0.05	0.70	0.52
Manic Cognitivity	D125-131	0.13	0.73	-0.07
Rhythmicity	D132-137	0.27	0.75	0.07
Rhythmicity	D138-149	0.65	0.46	-0.24
Rhythmicity	D150-161	0.67	0.40	0.09
Progesterone		0.24	0.09	0.87

The analysis of main components performed taking as items frequency of response to SCI-MOODS-SR domains and allopregnanolone concentrations, allows 3 components accounting for a total of 71.3% of variance to be identified. Table 5 illustrates the rotation matrix of components and reveals how component 1 (49.7% of total variance, Eigen value 6.0) aggregates SCI-MOODS-SR items: D1-28, Depressed Mood, (0.91); D58-67; Depressive Energy, (0.85); D81-98, Depressive Cognitivity, (0.74); D99-108, Depressive Cognitivity, (0.69); D150-161, Rhythmicity, (0.68), D138-149, Rhythmicity (0.67), D68-80; Manic Energy, (0.39); D29-57, Manic Mood (0.37); component 2 (12.4% of variance, Eigen value 1.5) aggregates SCI- MOODS-SR items: D132-137, Rhythmicity, (0.74); D68-80, Manic Energy, (0.74); D125-131, Manic Cognitivity, (0.73), D109-124, Manic Cognitivity (0.72); D29-57, Manic Mood, (0.71); D81-98, Depressive Cognitivity, (0.48); D138-149, Rhythmicity, (0.42) and D150-161, Rhythmicity, (0.39) and component 3 (9.2% of total variance, Eigen value 1.1) aggregates the level of allopregnanolone (0.87) with SCI-MOODS-SR items: D109-124, Manic Cognitivity, (0.50); D99-108, Depressive Cognitivity (0.34).

**Table 5:** Rotation matrix of SCI-MOODS-SR components/allopregnanolone (analysis of main components with Varimax rotation and Kaiser's normalization)

SCI-MOODS-SR DOMAIN	Item	Component 1	Component 2	Component 3
Depressed Mood	D1-28	0.91	0.19	0.08
Manic Mood	D29-57	0.37	0.71	0.12
Depressive Energy	D58-67	0.85	0.13	0.21
Manic Energy	D68-80	0.39	0.74	0.22
Depressive Cognitivity	D81-98	0.74	0.48	0.06
Depressive Cognitivity	D99-108	0.69	0.08	0.34
Manic Cognitivity	D109-124	-0.05	0.72	0.50
Manic Cognitivity	D125-131	0.15	0.73	-0.16
Rhythmicity	D132-137	0.28	0.74	0.06
Rhythmicity	D138-149	0.67	0.42	-0.20
Rhythmicity	D150-161	0.68	0.39	0.10
Allopregnanolone		0.25	0.07	0.87

The analysis of main components performed taking as items frequency of response to SCI-MOODS-SR domains and THDOC concentrations, allows 3 components accounting for a total of 70.4% of variance to be identified. Table 6 illustrates the rotation matrix of components and reveals how component 1 (33.4% of total variance, Eigen value 4.0) aggregates SCI-MOODS-SR items: D1-28, Depressed Mood, (0.92); D58-67, Depressive Energy (0.88); D81-98, Depressive Cognitivity. (0.75); D99-108, Depressive Cognitivity, (0.73); D150-161, Rhythmicity, (0.68); D138-149, Rhythmicity, (0.64); D68-80, Manic Energy (0.42); D29-57, Manic Mood (0.38); component 2 (27.2% of variance, Eigen value3.3) aggregates SCI- MOODS items D132-137, Rhythmicity, (0.76); D68-80, Manic Energy (0.74); D109-124, Manic Cognitivity, (0.74); D29-57, Manic Mood, (0.73); D125-131, Manic Cognitivity, (0.70); D81-98, Depressive Cognitivity (0.46); D138-149, Rhythmicity, (0.40); and D150-161, Rhythmicity, (0.37); and component 3 (9.8% of total variance, Eigen value 1.2) aggregates the level of THDOC (0.87) with SCI- MOODS items D109-124, Manic Cognitivity (0.39).

**Table 6:** Rotation matrix of SCI-MOODS-SR components/THDOC (analysis of main components with Varimax rotation and Kaiser's normalization)

SCI-MOODS-SR DOMAIN	Item	Component 1	Component 2	Component 3
Depressed Mood	D1-28	0.92	0.17	0.14
Manic Mood	D29-57	0.38	0.73	-0.07
Depressive Energy	D58-67	0.88	0.14	0.0.8
Manic Energy	D68-80	0.42	0.74	0.12
Depressive Cognitivity	D81-98	0.75	0.46	0.06
Depressive Cognitivity	D99-108	0.73	0.13	-0.09
Manic Cognitivity	D109-124	0.01	0.74	0.39
Manic Cognitivity	D125-131	0.15	0.70	0.03
Rhythmicity	D132-137	0.28	0.76	-0.12
Rhythmicity	D138-149	0.64	0.40	-0.13
Rhythmicity	D150-161	0.68	0.37	0.14
THDOC		0.07	0.06	0.96

Table 7 presents a summary of the SCI-MOODS-SR items which aggregate in each specific "3 component" deriving from analysis of the main components; it can be observed how allopregnanolone and progesterone aggregate with the "manic cognitivity" domain and with the "depressive cognitivity" sub-domain pertaining to suicidal ideas, while THDOC aggregates only with the former domain and cortisol only with the latter.

**Table 7:** Summary of SCI-MOODS-SR items which aggregate with neurosteroids in each specific "3 component" deriving from analysis of the main components.

	Depressive Cognitivity (Suicide)	Manic Cognitivity
Cortisol	Yes	No (weak)
Progesterone	Yes	Yes
Allopregnanolone	Yes	Yes
THDOC	No	Yes

## Discussion

Analysis of the main components at all domains of the SCI-MOODS-SR rating scale, together with the levels of cortisol, allopregnanolone, progesterone and THDOC (each of which introduced singly for a total of four analyses) underlines 3 components, of which the first 2, pertaining solely to SCI-MOODS-SR items, remain substantially homogeneous throughout the four analyses performed for each specific steroid.

The first component is the most representative in terms of general variability and refers to depressive symptoms (SCI-MOODS-SR domains D1-28 Depressed Mood; D58-67 Depressive Energy; D81-98, D99-108 Depressive Cognitivity; D150-161, D138-149 Rhythmicity) but some manic components are also correlated to it (both Manic Energy [D68-80] and Manic Mood [D29-57] are related by more than r > 0.30 in each of the four analysis). The second component refers to manic symptoms and aggregates with SCI-MOODS-SR domains D68-80 Manic Energy; D132-137 Rhythmicity; D125-131, D109-124 Manic Cognitivity; D29-57 Manic Mood; D81-98 Depressive Cognitivity (also this component showed some "mixed" elements).

The presence of components not associated with "pure" manic or depressive syndromes seems to confirm the validity of a unitary approach in the mood spectrum suggested by the findings of a study by Cassano and coll. [8]. This study showed a substantial number of manic/hypomanic symptoms in patients with recurrent unipolar depression and indicated that in both patients with recurrent unipolar depression and patients with bipolar I disorder the number of manic/hypomanic items of SCI-MOODS interview was related to an increased likelihood of endorsing suicidal ideation. According to the above cited results, our findings seem to challenge the traditional unipolar-bipolar dichotomy and bridge the gap between these two categories.

Introduction of the "neuroactive steroid" item does not modify the expected "core" profiles for mood disorders, depressive component 1 and manic component 2, both with a "mixed" component, but rather introduces a new exclusive component. Ultimately, depression or mania are the main syndromic components which correlate with neurosteroid concentrations, although manic components such as delusions of grandeur and, to a lesser but statistically significant degree, suicidal thoughts may also concur. The latter profile is clearly distinguishable for progesterone and allopregnanolone, cortisol is characterised by a tendency to correlate with manic components (whilst it actually correlates with depression) and THDOC correlates with manic ideas alone, rendering interpretation of this finding rather complicated. Should the hypothesis of a relationship between blood serum concentrations and clinical improvment of depressive symptomatology be confirmed, it may provide an explanation for the association with manic ideas, although it would not justify the association (albeit slight) with depressive ideas, particularly with the series of SCI-MOODS-SR items concerned with suicidal thoughts.

Similar to antidepressants, neurosteroids may possibly associate with improvement of syndromic components of "pure" forms of depression, but not with improvement of "mixed"states in which components such as suicidal ideas and delusions of grandeur frequently co-exist [21].

With regard to blood serum concentrations of cortisol, the possible existence of two distinct psychoneuroendocrinological profiles for depressive disorders has been hypothesized [22], the first characterized by hypercortisolemia and depressive melancholy, the second by hypocortisolemia and atypical depression.

The findings of the present study do not provide evidence to support this hypothesis; however, on assuming the presence of marked suicidal ideas in melancholy depression, it is feasible to maintain that a higher frequency of increased cortisol levels may have determined an association between this steroid and suicidal ideas. Moreover, atypical forms of depression would appear to be more frequent among subjects with bipolar disorders [23] and unlike allopregnanolone and progesterone, cortisol does not present higher levels in BD than in MDD subjects.

### Study limitations

#### Small sample size

The SCI-MOODS is an instrument that explores lifetime symptoms, while the neurosteroids concentrations are cross-sectional. As a consequence, the neurohormonal levels found performing the study 7 to 10 days before menstruation (a single observation per woman) are linked with syndromal clusters of lifetime mood spectrum. A study testing more than a single menstrual phase during a longer observational period should be carried out.

## Conclusion

Levels of allopregnanolone and progesterone do not correlate with association of the depressive and manic syndromes, but rather with "mixed" symptomatological aspects, and in particular with cognitive manic and depressive (with suicidal thoughts) dimensions.

The specific symptomatological areas involved may indeed suggest the involvement of an endogenous mechanism similar, at least in part, to that implicated for antidepressive drugs. The data obtained in our study do not appear to exclude the possibility that an increase in neurosteroid levels may be related to improvement of the clinical picture.

Further studies should be carried out to confirm these findings and to ascertain whether fluctuations observed in neurosteroid levels may be in some way related to the cyclicity of the bipolar spectrum, particularly to the prevailing of several syndromic components. To this regard, studies aimed at further investigating the role of neurosteroids in mood disorders could lead to the opening of new therapeutic frontiers.

## Authors' contributions

MCH, LDO and MGC conceived of the study, and participated in its design and coordination. CS and MGC participated in the design of the study and performed the statistical analysis. MCH, LDO and MGC drafted the manuscript. All authors read and approved the final manuscript.

## References

[B1] Biggio G Purdy RH Neurosteroids and Brain Function. International Review of Neurobiology 2001 46 San Diego: Academic Press

[B2] Smith SS Neurosteroid effects in the central nervous system Methods & New Frontiers in Neuroscience 2004 New York: CRS Press

[B3] Hardoy MC Serra M Carta MG Contu P Pisu MG Biggio G Increased neuroactive steroid concentrations in women with bipolar disorder and major depressive disorder J Clin Psychopharmacol 2006 26 379 84 10.1097/01.jcp.0000229483.52955.ec 16855455

[B4] Uzunova V Sheline Y Davis JM Rasmusson A Uzunov DP Costa E Guidotti A Increase in the cerebrospinal fluid content of neurosteroids in patients with unipolar major depression who are receiving fluoxetine or fluvoxamine Proc Natl Acad Sci USA 1998 95 3239 3244 9501247 10.1073/pnas.95.6.3239 PMC19726

[B5] George MS Guidotti A Rubinow D Pan B Mikalauskas K Post RM CSF neuroactive steroids in affective disorders: pregnenolone, progesterone, and DBI Biol Psychiatry 1994 35 775 780 10.1016/0006-3223(94)91139-8 8043707

[B6] Akiskal HS Bitar AH Puzantian VR Rosenthal TL Walker PW The nosological status of neurotic depression: a prospective three- to four-year follow-up examination in light of the primary-secondary and unipolar-bipolar dichotomies Arch Gen Psychiatry 1978 35 6 756 66 655773 10.1001/archpsyc.1978.01770300098011

[B7] Cassano GB Savino M Perugi G Musetti L Akiskal HS Major depressive episode: unipolar and bipolar II Encephale 1992 18 1 15 18 1600898

[B8] Cassano GB Rucci P Frank E Fagiolini A Dell'Osso L Shear M Kupfer DJ The mood spectrum in unipolar and bipolar disorder: arguments for a unitary approach Am J Psychiatry 2004 161 1264 1269 10.1176/appi.ajp.161.7.1264 15229060

[B9] Akiskal HS Benazzi F Toward a clinical delineation of dysphoric hypomania -operational and conceptual dilemmas Bipolar Disord 2005 7 5 456 464 10.1111/j.1399-5618.2005.00242.x 16176439

[B10] Angst J Cassano GB The mood spectrum: improving the diagnosis of bipolar disorder Bipolar Disord 2005 7 Suppl 4 4 12 10.1111/j.1399-5618.2005.00210.x 15948762

[B11] American Psychiatric Association Diagnostic and statistical manual of mental disorders, text revision (DSM-IV-TR) 2000 4 Washington DC

[B12] Purdy RH Morrow AL Blinn JR Paul SM Synthesis, metabolism, and pharmacological activity of 3α-hydroxy steroids which potentiate GABA receptor-mediated chloride ion uptake in rat cerebral cortical synaptoneurosomes J Med Chem 1990 33 1572 1581 10.1021/jm00168a008 2160534

[B13] Barbaccia ML Roscetti G Trabucchi M Mostallino MC Concas A Purdy RH Biggio G Time dependent changes in rat brain neuroactive steroid concentrations and GABAA receptor function after acute stress Neuroendocrinol 1996 63 2 166 172 9053781 10.1159/000126953

[B14] First MB Spitzer RL Gibbon M Williams JBW Structured Clinical Interview for DSM-IV Axis I Disorders (SCID-I) American Psychiatric Publishing Inc 1997

[B15] American Psychiatric Association Diagnostic and statistical manual of mental disorders, (DSM-IV) 1994 4 Washington DC

[B16] Cassano GB Dell'Osso L Frank E Miniati M Fagiolini A Shear K Pini S Maser J The bipolar spectrum: a clinical reality in search of diagnostic criteria and an assessment methodology J Affect Disord 1999 54 319 328 10.1016/S0165-0327(98)00158-X 10467978

[B17] Fagiolini A Dell'Osso L Pini S Armani A Bouanani S Rucci P Cassano GB Endicott J Maser J Shear MK Grochocinski VJ Frank E Validity and reliability of a new instrument for assessing mood symptomatology: the Structured Clinical Interview for Mood Spectrum (SCI-MOODS) Int J Meth Psych Res 1999 8 71 81 10.1002/mpr.58

[B18] Cassano GB Frank E Miniati M Rucci P Fagiolini A Pini S Shear MK Maser JD Conceptual underpinnings and empirical support for the mood spectrum Psychiatr Clin North Am 2000 25 4 699 712 10.1016/S0193-953X(02)00025-4 12462856

[B19] Cassano GB Pini S A spectrum model for mood disorders: a useful approach in clinical psychiatry in search of an assessment methodology Epidemiol Psychiatr Soc 2002 9 156 162 10.1017/s1121189x00007843 11094837

[B20] Dell'Osso L Armani A Rucci P Frank E Fagiolini A Corretti G Shear MK Grochocinski VJ Maser JD Endicott J Cassano GB Measuring mood spectrum: comparison of interview (SCI-MOODS) and self-report (MOODS-SR) instruments Compr Psychiatry 2002 43 1 69 73 10.1053/comp.2002.29852 11788923

[B21] Akiskal HS Benazzi F Psychopathologic correlates of suicidal ideation in major depressive outpatients: is it all due to unrecognized (bipolar) depressive mixed states? Psychopathology 2005 38 5 273 280 10.1159/000088445 16179814

[B22] Gold PW Crousos GP Organization of the stress system and its dysregulation in melancholic and atypical depression: high vs low CRH/NE states Mol Psychiatry 2002 7 3 254 257 10.1038/sj.mp.4001032 11920153

[B23] Fountoulakis KN Iacovides A Fotiou F Nimatoudis J Bascialla F Ioannidou C Kaprinis G Bech P Neurobiological and psychological correlates of suicidal attempts and thoughts of death in patients with major depression Neuropsychobiology 2004 49 1 42 52 10.1159/000075338 14730200

